# Neurobehavioral Performance in Preschool Children Exposed Postnatally to Organophosphates in Agricultural Regions, Northern Thailand

**DOI:** 10.3390/toxics12120855

**Published:** 2024-11-27

**Authors:** Ajchamon Thammachai, Boonsita Suwannakul, Noppharath Sangkarit, Surat Hongsibsong, Juthasiri Rohitrattana, Ratana Sapbamrer

**Affiliations:** 1 Department of Physical Therapy, School of Allied Health Sciences, University of Phayao, Phayao 56000, Thailand; ajchamon@gmail.com (A.T.); boonsita.su@up.ac.th (B.S.); noppharath.sa@up.ac.th (N.S.); 2 School of Health Sciences Research, Research Institute for Health Sciences, Chiang Mai University, Chiang Mai 50200, Thailand; s_hongsibsong@hotmail.com; 3 Center for Safety, Health and Environment, Chulalongkorn University, Bangkok 10330, Thailand; juthasiri.r@gmail.com; 4 Department of Community Medicine, Faculty of Medicine, Chiang Mai University, Chiang Mai 50200, Thailand; 5 Environmental and Occupational Medicine Excellence Center (EnOMEC), Faculty of Medicine, Chiang Mai University, Chiang Mai 50200, Thailand

**Keywords:** children, pesticide, neurobehavior, neurodevelopment, organophosphate

## Abstract

Evidence of the effects of postnatal exposure to organophosphates (OPs) on children’s neurodevelopment remains limited but crucial. This cross-sectional study evaluated exposure to OPs and neurobehavioral performance in 172 preschool children. Urinary dialkyl phosphate (DAP) metabolites, biomarkers for exposure to OPs, were measured. The neurobehavioral assessments included motor skills, memory, and cognitive function, measured using the Purdue pegboard test, digit span test, object memory test, and visual-motor integration. Multiple linear regression models were employed to explore the associations between urinary DAP metabolite levels and neurobehavioral performance, adjusting for potential confounders. Findings revealed that children of farming parents had higher urinary levels of dimethylphosphate (DMP) (Beta = 0.730, 95% CI = 0.138, 1.322, *p* value = 0.016) and diethylphosphate (DEP) (Beta = 0.668, 95% CI = 0.044, 1.291, *p* value = 0.036). Additionally, high fruit consumption correlated with increased urinary DEP levels (Beta = 0.398, 95% CI = 0.063, 0.733, *p* value = 0.020). Critically, elevated urinary DEP was associated with poorer fine motor coordination, affecting performance in the Purdue pegboard test for the dominant hand (Beta = −0.428, 95% CI = −0.661, −0.194, *p* value < 0.001), the preferred hand (Beta = −0.376, 95% CI = −0.603, −0.149, *p* value = 0.001), and both hands (Beta = −0.524, 95% CI = −0.773, −0.276, *p* value < 0.001). These findings highlight the role of parental occupation and diet in children’s OP exposure and suggest that OP exposure negatively impacts fine motor coordination. Targeted interventions, such as promoting organic diets, enhancing workplace safety, and ongoing biomonitoring, are vital to reduce neurodevelopmental risks for vulnerable populations.

## 1. Introduction

Organophosphates (OPs) are widely used in agricultural settings to control pests and increase crop yields. However, exposure to these chemicals poses significant health risks, particularly for young children, whose developing nervous systems are highly vulnerable to environmental toxicants [1,2]. Thailand is one of the largest agricultural producers in Southeast Asia, with a significant portion of its economy reliant on farming [3]. Northern Thailand, in particular, is a critical agricultural region, known for its mountainous terrain and fertile valleys, where intensive farming activities dominate [4]. Crops are typically grown in close proximity to rural communities, increasing the likelihood of pesticide exposure among local residents, including children [5,6]. In rural agricultural communities, children are often exposed to OPs through multiple pathways. These include direct exposure via their parents’ occupational activities, as pesticide residues can be transferred from the workplace to the home environment, and indirect exposure through diet, particularly the consumption of fruits and vegetables [7,8,9]. Additionally, playing habits of children, such as spending time on or near farmland, can further increase their risk of exposure [7,10,11].

Neurodevelopmental health in children, especially those in rural agricultural areas, is a growing concern. Early childhood is a period of rapid brain development, during which children are highly sensitive to environmental toxicants [2,12]. While many studies have confirmed the harmful effects of prenatal exposure to OPs on child neurodevelopment, there remains controversy about the different impacts of prenatal versus postnatal exposure [13,14,15]. A systematic review by Sapbamrer and Hongsibsong [13] found that prenatal exposure to OPs significantly contributes to neurodevelopmental disorders, including motor and cognitive deficits in children. In contrast, data on postnatal exposure are limited and less consistent. Although few studies have examined postnatal effects, they suggest potential links to neurodevelopmental challenges, particularly with chronic, low-dose exposure. Postnatal exposure may contribute to cumulative neurodevelopmental damage, especially in children aged 4–5 years, a critical period for brain development and environmental interaction [7,10]. More standardized research is needed to confirm these associations and draw clearer conclusions.

This study aims to address these gaps by investigating the association between postnatal exposure to OPs and neurobehavioral performance in preschool children living in agricultural communities in northern Thailand. Urinary dialkyl phosphate (DAP) metabolites, biomarkers for exposure to OPs, were measured. The neurobehavioral assessments included motor skills, memory, and cognitive function, measured using the Purdue pegboard test, digit span test, object memory test, and visual-motor integration. Focusing on this age group helps us understand the potential long-term impacts of early-life exposure on neurodevelopment. We also examine factors associated with exposure to OPs among children. The findings from this study will provide valuable insights into the neurodevelopmental risks associated with postnatal exposure to OPs and offer evidence to inform targeted interventions and policies aimed at reducing pesticide exposure in vulnerable populations, ultimately contributing to healthier developmental outcomes for children in agricultural communities.

## 2. Materials and Methods

### 2.1. Settings and Population

Phayao province, in northern Thailand, is predominantly agricultural, with widespread use of insecticides. According to a preliminary survey, agricultural activities in the region generally begin in January and extend through the harvest season in June. During this period, there is substantial pesticide application, with OPs being the most commonly used. This cross-sectional study was conducted between January and June 2024 to investigate exposure to OPs and neurobehavioral performance among preschool children living in agricultural communities in Phayao province, northern Thailand. The research aimed to identify factors associated with urinary levels of DAP metabolites and evaluate their potential effects on children’s neurobehavioral performance.

The study population consisted of preschool children living in agricultural communities in Phayao province, northern Thailand. We focused on preschool children because their behaviors, such as hand-to-mouth activities and increased contact with the environment, along with consuming more varied diets than infants and toddlers, increase their likelihood of exposure to toxicants. Additionally, their detoxification systems are still developing, making it more difficult for them to process harmful substances. Studying this age group provides valuable insights into how early-life exposures may impact long-term neurodevelopmental outcomes. The inclusion criteria included the following: (i) children aged 4–5 years; (ii) children who had resided in these agricultural communities for more than one year; (iii) children in good health; (iv) parents or guardians of Thai nationality; (v) parents living in the same household as the children; and (vi) both the children and their parents expressing willingness to participate in the study. Exclusion criteria included the following: (i) children with a prior diagnosis of developmental or neurological disorders or chronic conditions such as liver, kidney, or lung disease, diabetes, cancer, or serious communicable diseases; (ii) children whose parents had a history of neurological conditions such as Alzheimer’s disease, Parkinson’s disease, or diabetes, as well as other conditions including mental health disorders or thyroid disease; and (iii) children whose parents were unable to read, write, or comprehend the Thai language. Children were recruited from agricultural communities using a systematic approach. Eligible children were identified through local registry records maintained by community health promotion hospitals in the study area. Trained field researchers conducted home visits to provide detailed information about the study, obtain informed consent, and recruit participants. This recruitment strategy ensured the inclusion of children actively engaged in agricultural activities, enhancing the relevance of the findings to this vulnerable population. Parents were required to provide written informed consent, signifying their agreement to allow their children to participate in the study. If a child demonstrated unwillingness or discomfort on the scheduled testing day, such as by showing fear or crying, the assessment was promptly halted and postponed to a later date when the child was more cooperative and comfortable with the testing process.

Of the 190 parent–child pairs who met the inclusion criteria and provided parental consent for participation, 18 children (9.5%) were excluded from the study due to difficulties in collecting urine samples and assessing neurobehavioral performance. Consequently, the final sample comprised 172 parent–child pairs. This study was conducted with approval from the Research Ethics Committee of the Faculty of Medicine, Chiang Mai University, Thailand (Approval No. 129/2023, approved on 3 April 2023).

### 2.2. Interviews

The parents were interviewed using a structured questionnaire to collect information about both themselves and their children (parent—child pair). In this study, “parents” are defined as fathers, mothers, legal guardians, or family members who were the primary caregivers, residing in the same household as the children and responsible for their care and upbringing. The data gathered on the parents included the following: age (in years), gender (male or female), educational level (primary school or below, secondary school, or bachelor’s degree or higher), monthly income (<150 USD, 150–300 USD, or >300 USD), occupation (farmer or non-farmer), smoking status (yes or no), alcohol consumption (yes or no), proximity of the household to farmland (<0.5 km, 0.5–2 km, or >2–5 km), and relationship to the primary caregiver (father/mother or other). Information collected on the children included age (in months), gender (male or female), body mass index (BMI), playing on farmland (yes or no), frequency of vegetable and fruit consumption (never, rarely (1–2 servings/week), often (3–5 servings/week), or always (6–7 servings/week)), and usual place for purchasing vegetables and fruits (local markets or supermarkets). The interviews were conducted by trained interviewers and took approximately 10–15 min to complete.

### 2.3. Measurement of Urinary DAP Metabolites

Morning voided urine samples (100–150 mL) were collected from children to measure the levels of DAP metabolites. The urine samples were transported to the laboratory in iceboxes and stored at −20 °C until analysis. DAP metabolites are indicators of exposure to OPs, including dimethylphosphate (DMP), dimethylthiophosphate (DMTP), dimethyldithiophosphate (DMDTP), diethylphosphate (DEP), diethylthiophosphate (DETP), and diethyldithiophosphate (DEDTP). Extraction and analysis were performed according to the method of Prapamontol et al. [16], using gas chromatography (Hewlett Packard 6890-FPD, Agilent Technology, Santa Clara, CA 95051, USA). The conditions were as follows: Column: Agilent HP-5 (30 m × 0.25 mm i.d., 0.25 µm film thickness); Oven temperature program: (1) Initial temperature set at 100 °C, increased at a rate of 30 °C/min to 180 °C, held for 3.55 min; (2) Further increased at 30 °C/min to 260 °C, held for 1 min; (3) Post-run temperature set to 290 °C for 2 min; Detector temperature: 250 °C; Inlet pressure: 27 psi; Injection volume: 1 µL; Carrier gas: Helium (99.99% purity). The levels of DAP metabolites were normalized to creatinine concentrations and reported as µg/g creatinine. Creatinine levels were determined using the Jaffe reaction (colorimetric method). Samples with concentrations below the limit of detection (LOD) were assigned a value of LOD divided by the square root of 2, as recommended by Hornung and Reed [17].

Quality control was conducted to ensure the accuracy and reliability of metabolite measurements in the study. The results showed that the LOD ranged from 0.17 µg/L for DMDTP to 1.40 µg/L for DETP, while the limits of quantification (LOQ) spanned from 0.57 µg/L for DMDTP to 4.65 µg/L for DETP. The recoveries were between 94.46% for DMTP and 108.4% for DETP. The intra-batch coefficient of variation (%CV) ranged from 4.14% for DMP to 11.13% for DMDTP, while the inter-batch %CV varied from 6.59% for DMP to 9.58% for DMDTP.

### 2.4. Neurobehavioral Tests

The neurobehavioral tests used in this study were originally part of the Behavioral Assessment and Research System (BARS) [18,19]. These tests were translated into Thai and piloted by a research team before being implemented in earlier research [20]. Specifically, Juthasiri Rohitrattana, one of the research team, adapted these assessments for use with Thai children [20]. The four non-computerized neurobehavioral tests employed included the Purdue pegboard test (PEG), digit span test (DST), object memory test (OMT), and visual-motor integration (VMI). The test–retest reliabilities for the tests without alternate forms ranged from 0.41 to 0.77.

The PEG test is a well-established neuropsychological tool to evaluate motor dexterity, hand–eye coordination, and the ability to perform tasks requiring precise finger movements. In this study, the Purdue pegboard Model 32020A (Lafayette Instrument Company, Lafayette, IN 47904, USA) was used. The device consists of two vertical rows of 25 small holes running down the center of the board, with four cups positioned at the top. Two of the outer cups contain 25 pins. During the test, participants were tasked with placing small pegs into the holes within a 30 s time frame for each trial. The number of pegs placed was recorded for the preferred hand, non-preferred hand, and both hands. Each subtest was conducted twice per individual, and the average number of pegs placed across the two trials was used to determine the final score for each subtest. The maximum score for each subtest was 25.

The DST is a commonly used neuropsychological assessment that measures short-term memory, working memory, and attention. Participants are presented with a sequence of digits and instructed to recall them in the same order (forward span). To minimize repetition, the number sequences were pseudo-randomized. The forward digit span task requires participants to repeat the digits in the same sequence as presented. The investigator pronounces a series of digits at a rate of approximately one per second, and the participant is tasked with repeating the sequence. The length of the digit sequences progressively increases, starting with a two-number sequence and extending up to nine digits. Two different sets of forward digit span tasks were administered in Trial 1 and Trial 2, with Trial 1 completed before Trial 2 to assess cognitive flexibility. The raw scores for the forward digit span were represented by five distinct values, including the total number of correct responses from both trials. The maximum digit length achieved by each participant—the longest sequence they could accurately recall—was also recorded as the maximum digit forward. The total possible score for the forward digit span task was 16, while the maximum digit span score was 9.

The OMT test is designed to evaluate memory and recognition skills. In this test, participants are shown a set of common objects that they are asked to remember. After a brief interval, they are tested on how many objects they can recall (immediate recall) and again after a longer delay (delayed recall). Participants are also asked to recognize these objects from a larger group of distractor items, tested on the recognition of the target and non-target items (recognition memory). Scores were recorded for immediate recall, delayed recall, and recognition, with a maximum score of 16 for each measure.

The VMI test assesses the coordination between visual perception and motor skills, specifically the ability to convert visual information into precise motor actions. In this study, the Beery-Buktenica Developmental Test of Visual-Motor Integration, Sixth Edition (VMI-6)-short form, was used as the assessment tool [21,22]. Participants were instructed to replicate or draw 21 geometric shapes they observed, requiring the integration of visual perception with fine motor control to accurately reproduce the stimuli. One point is scored for each correctly copied item, while no points are given for items that do not meet the manual’s criteria. The ceiling score is determined after three consecutive forms are not passed. Results are presented as both raw and standardized scores, following a summary calculation of each.

All assessments were administered by trained examiners who had undergone instruction from experienced investigators and psychologists. The test–retest reliability correlation coefficients for the various assessments among children were as follows: 0.71 to 0.72 for the PEG, 0.41 to 0.48 for the DST, 0.16 to 0.35 for the OMT, and 0.64 for the VMI test [20]. These reliability measures reflect the consistency of performance across repeated testing sessions.

### 2.5. Data Analysis

Descriptive statistics, including mean, geometric mean (GM), median, percentiles, percentage (%), minimum (Min), maximum (Max), and standard deviation (SD.) were computed to summarize the socio-demographic characteristics, pesticide exposure levels, and neurobehavioral performance of the study population. Missing data for continuous variables were imputed using the mean value, while missing categorical data were imputed using the mode. This approach ensured that all available data were utilized, minimizing the impact of missing values on the overall analysis without introducing significant bias. Urinary DAP metabolite levels were normalized to creatinine and expressed in µg/g creatinine. GM and detection frequencies were calculated for each DAP metabolite as well as for total DAP levels. Given that urinary DAP metabolite levels exhibited skewed distributions, logarithmic transformations (Ln) were applied to normalize the data and reduce the influence of outliers.

Multiple linear regression models were used to investigate the relationships between urinary DAP metabolite levels and potential predictors. Covariates for inclusion in the regression models were selected based on a well-established theoretical framework aligned with the research objectives and hypotheses and further supported by a correlation coefficient threshold of *p* value < 0.2. This threshold was selected to ensure that potential confounders with weaker associations were not prematurely excluded, as is common in exploratory epidemiological analyses. Key predictors included parental factors (age, gender, smoking status, occupation, and proximity of the household to farmland) and child-related factors (gender, BMI, playing on farmland, and frequency of vegetable and fruit consumption). Additionally, linear regression models were used to examine associations between urinary DAP metabolite levels and neurobehavioral outcomes. Confounders were selected using the same theoretical framework and correlation threshold (*p* value < 0.2). Confounders included parental factors (age, gender, education, income, smoking status, and alcohol consumption) and child-related factors (age, gender, and BMI). For all regression models, beta coefficients and 95% confidence intervals (CI) were reported to quantify the strength and direction of the associations. A significance level of *p* value < 0.05 was applied to all statistical tests to determine statistical significance.

## 3. Results

### 3.1. Socio-Demographic Characteristics and Pesticide Exposure

The study included 172 parents and 172 preschool children. Among the parents, 32% were male and 68% were female, with an average age of 50.1 ± 12.4 years. The majority (66.3%) had a monthly income of less than 150 USD, and most had an education level of primary school or less (52.9%). A significant portion (82%) were engaged in farming, and 19.2% of parents reported smoking. Additionally, 44.2% consumed alcohol. A majority of households (87.2%) were located more than 500 m from farmland.

Among the children, the average age was 4.4 ± 0.5 years, with 45.3% being male and 54.7% female. The children’s mean BMI was 15.6 ± 2.7. About half of the children (50%) frequently played on farmland, and a significant proportion (54.1%) rarely consumed vegetables, while 59.3% often consumed fruits (Table 1).

### 3.2. Urinary Levels of DAP Metabolites

The detection of urinary DAP metabolites among preschool children varied. The GM for total DMP was 7.43 µg/g creatinine, and the total DEP was 10.51 µg/g creatinine, with the total DAP detection rate at 67.4%. The highest detected metabolite was DEP (38.4%), while DEDTP had the lowest detection rate (1.2%) (Table 2).

### 3.3. Neurobehavioral Performance

The neurobehavioral performance of the children was assessed using various tests. The mean PEG scores for dominant hand, preferred hand, and both hands were 7.48 ± 1.62, 6.85 ± 1.56, and 5.52 ± 1.78, respectively. The DST revealed an average forward span of 7.03 ± 1.98 and a forward maximum of 4.79 ± 1.17. The OMT scores for immediate recall, delayed recall, and recognition were 14.64 ± 1.32, 14.85 ± 1.18, and 14.03 ± 1.60, respectively. The VMI raw and standard scores were 11.48 ± 3.19 and 99.79 ± 14.06, respectively (Table 3).

### 3.4. Factors Associated with Urinary DAP Metabolites in Preschool Children

Multiple linear regression analysis identified several factors associated with urinary DAP metabolite levels in preschool children. Parental farmer status was significantly associated with increased urinary DMP levels (Beta = 0.730, 95% CI = 0.138, 1.322) and DEP levels (Beta = 0.668, 95% CI = 0.044, 1.291). Children with higher BMI had significantly lower urinary DMP levels (Beta = −0.071, 95% CI = −0.139, −0.004). The frequency of fruit consumption was positively associated with urinary DEP levels (Beta = 0.398, 95% CI = 0.063, 0.733) (Table 4).

### 3.5. Association Between Urinary DAP Metabolites and Neurobehavioral Performance

Higher urinary levels of total DEP metabolites were significantly associated with poorer performance in the PEG score, specifically in the dominant hand (Beta = −0.428, 95% CI = −0.661, −0.194), preferred hand (Beta = −0.376, 95% CI = −0.603, −0.149), and both hands (Beta = −0.524, 95% CI = −0.773, −0.276) (Table 5).

## 4. Discussion

In this study, the variability in urinary DAP metabolite levels among preschool children underscores exposure to OPs in northern Thailand’s agricultural communities. The detection of DAP metabolites in 67.4% of urine samples, with the DEP group being the most frequently detected (60.5%), suggests that OPs in the DEP group are a predominant marker of exposure to OPs in this population. These findings are consistent with other studies in similar rural agricultural settings, where exposure to OPs is prevalent [10,23,24]. The GM concentrations of total DMP (7.43 µg/g creatinine) and total DEP (10.51 µg/g creatinine) also provide valuable insights into the exposure patterns. The higher levels of DEP, in particular, may reflect the common use of OPs that metabolize into DEP. Among the numerous pesticides imported into Thailand in 2023 were the OPs categorized under the DEP group, such as diazinon, ethion, prothiophos, and phenthoate [25]. Additionally, farmers at the study site commonly and extensively used OPs classified under the DEP group, including chlorpyrifos, profenofos, and triazophos. It further underscores the need for monitoring and regulating the use of these pesticide groups in such environments.

In this study, we investigated various parental and child factors associated with urinary DAP metabolites, specifically total DMP and total DEP, among preschool children in an agricultural community. Multiple linear regression analysis identified several key factors with significant associations. Among parental factors, being a farmer was significantly associated with higher urinary levels of both total DMP and total DEP, suggesting that parental occupational exposure plays a critical role in influencing children’s exposure to OPs. This is consistent with previous studies demonstrating the influence of parental occupational pesticide exposure on DAP metabolite levels in their children’s urine [26]. These findings highlight the potential for take-home exposure, where pesticides are transported from the workplace to the home environment, increasing children’s risk. Take-home exposure can occur through contaminated clothing, skin, shoes, or equipment, leading to secondary exposure for household members, particularly children, who may be more vulnerable due to their developing physiology. In agricultural communities where OPs use is common, the risk of take-home exposure is significant, as farmers often work in close contact with these chemicals and may unintentionally bring pesticide residues into their homes [27,28]. Available studies indicate that higher levels of OP residues in house dust have been found in the homes of agricultural workers [29,30,31]. A systematic review by López-Gálvez et al. [32] also indicates that farmworker families experience higher pesticide exposure levels compared to non-farmworker families.

Several studies have documented that children living in agricultural communities tend to have higher urinary levels of DAP metabolites compared to those in non-agricultural communities [10,14]. This suggests that the home environment, as an extension of the occupational setting, becomes a key site of exposure. Children are particularly susceptible due to behaviors such as hand-to-mouth activity, floor play, and proximity to surfaces that may be contaminated [7,10]. In addition, the detoxification system in young children may be immature and under-functioning, leading to prolonged pesticide exposure compared to adults [33,34]. To effectively eliminate take-home exposures and protect the health of workers’ families and communities, a comprehensive, multi-tier prevention strategy is essential. This should include stricter enforcement of workplace safety standards and hygiene protocols, educating workers and their families on reducing home contamination through improved hygiene practices, and implementing local health education programs and interventions. Protective measures, such as changing clothes and showering before returning home, can significantly reduce the transfer of pesticides. Additionally, cleaning contaminated work items, such as shoes and equipment, outside the living area is crucial. Regular cleaning of home surfaces, especially in areas where children play, further minimizes the risk of exposure. Strengthening the enforcement of safety regulations, increasing support for workers, and adopting an eco-social framework that addresses the broader social and economic drivers of exposure risks are also vital. Community empowerment, with a focus on including vulnerable groups, is emphasized, along with the need for inter-agency collaboration and data integration to enhance monitoring and effectively tackle this issue [27,28].

Another notable finding was the association between children’s BMI and urinary DMP. Children with lower BMI exhibited significantly higher urinary total DMP levels, suggesting that body composition may influence the absorption, distribution, or metabolism of OPs. These results align with a previous study that found higher urinary levels of DAP metabolites were associated with lower BMI and waist circumference [35]. Potential mechanisms include decreased appetite and altered gastrointestinal function due to exposure to OP. Additionally, some OPs may accumulate in fatty tissues, affecting the excretion and body weight relationship [35]. Our findings also revealed a significant positive association between frequent fruit consumption and urinary total DEP levels, suggesting that fruits may be a key dietary source of pesticide exposure. Conversely, no significant association was found between vegetable consumption and DAP metabolite levels, which may be explained by differences in pesticide application across crops or regional agricultural practices. It is likely that diet, particularly fruit consumption, plays a pivotal role in children’s exposure to OPs [8]. Prior research in northern Thailand indicated that OP residues from the DEP group, including chlorpyrifos, diazinon, triazophos, and ethion, were frequently detected in both fruits and vegetables [24,36]. Notably, residue levels of OPs in fruits such as oranges, pears, and red grapes often exceeded the Maximum Residue Limits (MRLs) set by Codex Thailand and Codex WHO/FAO [36]. Furthermore, the research indicates that most children prefer sweet-tasting fruits over strongly flavored vegetables [37], which may contribute to increased risk of exposure to OPs. Interestingly, our analysis also identified a significant association between parental farmer status and children’s frequency of vegetable and fruit consumption (*p* value = 0.030 for vegetable consumption, and *p* value < 0.001 for fruit consumption) (Table S1). This finding suggests that farming households may directly provide greater quantities of fruits and vegetables to their families, potentially increasing both the nutritional benefits and pesticide exposure risks associated with these food items. The consumption of fruits and vegetables is widely recognized for its significant health benefits, including reducing the risk of chronic diseases and promoting overall well-being due to their high antioxidant content. However, concerns about pesticide residues associated with these foods cannot be overlooked. While fruits and vegetables are essential components of a balanced diet, particularly for children, dietary habits and the sources of produce can significantly influence pesticide exposure levels. To mitigate this risk, strategies such as consuming organic produce or thoroughly washing and peeling fruits and vegetables are strongly recommended. Research has shown that adopting an organic diet can effectively reduce urinary pesticide levels in children, making it a practical and impactful intervention. This effect has been consistently observed across diverse populations worldwide [38,39]. Additionally, our analysis also found no association between BMI and the frequency of fruit and vegetable consumption (*p* value = 0.623 for fruit consumption and *p* value = 0.532 for vegetable consumption) (Table S2). These findings suggest that BMI, as an indicator of nutritional status, in this study is not directly related to the frequency of fruits and vegetables.

Regarding the association between urinary DAP metabolites and neurobehavioral performance among children, our results reveal that elevated urinary total DEP metabolite levels are significantly associated with poorer performance on the PEG test, specifically in the dominant hand, preferred hand, and both hands. These findings suggest that heightened exposure to OPs, particularly those in the DEP group, may impair fine motor skills and coordination. The PEG test is a well-established metric for assessing motor dexterity and hand-eye coordination, both critical for daily activities and cognitive development in children. Our findings imply that exposure to OPs may disrupt neural pathways involved in motor control, aligning with previous research that highlights deficits in motor skills and coordination among children exposed to OPs [31,40,41]. The neurobehavioral deficits observed in children exposed to OPs are likely mediated by multiple mechanisms, influenced by the intensity and duration of exposure [42]. A primary mechanism involves the inhibition of acetylcholinesterase (AChE), an enzyme essential for breaking down acetylcholine (ACh) in synaptic clefts. The inhibition of AChE results in the accumulation of ACh, leading to overstimulation of cholinergic receptors in both the central and peripheral nervous systems. In the central nervous system, this excessive ACh interferes with normal neurotransmission, particularly in brain regions responsible for motor control and coordination. The inhibition of AChE within the basal forebrain cholinergic system, which regulates motor control and attention, may further contribute to impaired motor inhibition in children [41]. Additionally, overstimulation of muscarinic and nicotinic receptors can trigger neuropsychiatric symptoms, compounding the adverse effects on neurobehavioral performance, including fine motor skills and dexterity [42]. Moreover, exposure to OPs compromises peripheral nerve function. Acute OP poisoning has been shown to cause subclinical axonal damage and demyelination, affecting motor nerve conduction velocity and the amplitude of compound muscle action potentials. These physiological disruptions manifest as motor function impairments, such as muscle weakness and reduced coordination [43,44].

Children are particularly vulnerable to the neurotoxic effects of OPs due to the immaturity of their developing nervous systems and detoxification mechanisms compared to adults. Even at low levels, exposure to OPs during critical periods of neurodevelopment can interfere with essential neurotransmitter functions, particularly ACh, which plays a pivotal role in brain and cellular development [45]. Synapses are especially susceptible to exposure to OPs during this period, and alterations in synaptic function may contribute to the development of neurodevelopmental disorders [46]. Furthermore, exposure to OPs has been linked to oxidative stress and mitochondrial dysfunction. Mitochondrial impairment leads to oxidative stress and diminished adenosine triphosphate (ATP) levels, disrupting energy regulation, cell function, and eventually causing cell death. Oxidative stress and apoptosis are key factors in OP-induced neurodegeneration, causing axonal degeneration and cytoskeletal damage in peripheral nerves, consequently leading to motor function deficits [42,44,47,48]. However, it is noteworthy that no statistically significant associations were found between urinary DAP metabolite levels and performance on other neurobehavioral tests in our study. This disparity may be due to the distinct sensitivity of each test to OP-induced neurotoxicity. For instance, the PEG test emphasizes fine motor coordination, which may be more vulnerable to OP-related disruptions compared to cognitive tasks like the DST or VMI. Additionally, the intensity and duration of exposure could influence outcomes, with higher or more prolonged exposure potentially required to affect domains beyond motor dexterity. The timing of exposure is also critical, as fine motor coordination, which develops rapidly in early childhood, may be particularly susceptible during this period. Moreover, OP toxicity mechanisms, including acetylcholinesterase inhibition and oxidative stress, may selectively affect motor pathways more than other cognitive functions.

This study has several notable strengths and limitations. One strength is the comprehensive assessment of exposure to OPs using DAP metabolites, which provide a reliable measure of exposure. The inclusion of multiple neurobehavioral tests strengthens the study further by evaluating various cognitive and motor skills in children. Additionally, the focus on preschool children from agricultural communities offers valuable insights into an underrepresented and vulnerable population. The use of multiple linear regression analysis, adjusting for several confounders, enhances the robustness of the findings. However, this study also has limitations. Its cross-sectional design limits the ability to draw causal inferences between OP exposure and neurodevelopmental outcomes. A key limitation is that urinary DAP metabolite levels were measured from a single urine sample for each participant. While single-point sampling provides a snapshot of recent exposure, it may not accurately capture longer-term or chronic pesticide exposure due to the short half-life of DAP metabolites. This variability could reduce the ability to detect associations and may underestimate the relationship between OP exposure and neurodevelopmental outcomes. To mitigate this limitation, urine samples were collected during the peak pesticide use season in the study area to better reflect typical exposure levels. Additionally, we adjusted for potential confounders and interpreted the findings cautiously, recognizing the inherent variability in exposure measurement. Lastly, despite adjusting for key factors, there remains the potential for unmeasured confounders, such as other environmental toxicants or genetic susceptibilities, which could influence the results. Based on these limitations, several areas for future research warrant exploration. First, longitudinal studies are needed to establish causal relationships between exposure to OPs and neurodevelopmental outcomes. Future research should also aim to collect multiple urine samples over time to provide a more accurate reflection of chronic exposure to OPs, reducing variability due to short-term fluctuations in pesticide exposure. Additionally, studies conducted in diverse geographic regions with varying agricultural practices are necessary to assess the generalizability of the findings beyond northern Thailand. Such studies could also explore differences in dietary sources of exposure to OPs, considering the role of local agricultural practices and pesticide residues in food. Studies with larger sample sizes should be conducted to ensure sufficient statistical power for detecting smaller effect sizes in neurodevelopmental outcomes. The use of a Directed Acyclic Graph (DAG) offers a more theoretically robust approach to covariate selection by explicitly integrating prior knowledge of causal relationships and reducing the risk of over-adjustment or collider bias. Future research should consider employing DAGs to enhance the rigor and validity of analytical frameworks in similar studies. Finally, future research should investigate other potential environmental and genetic factors that may confound the relationship between exposure to OPs and neurodevelopment.

## 5. Conclusions

This study provides evidence that postnatal exposure to OPs, particularly through the DEP group, is linked to neurobehavioral impairments in preschool children, especially in fine motor coordination. Parental occupational exposure, particularly in farming, was a significant contributor to elevated urinary DAP levels in children. Dietary factors, such as frequent fruit consumption, were also associated with higher urinary DEP levels, indicating that diet plays a role in exposure to OPs. The findings highlight the need for comprehensive interventions that address both occupational and dietary sources of pesticide exposure in agricultural communities. These interventions should include behavioral changes, workplace safety improvements, and community-wide education and policy initiatives aimed at reducing pesticide contamination in both work and home environments. Continuous biomonitoring of pesticide exposure in vulnerable populations, particularly young children, is crucial for mitigating potential neurodevelopmental risks. Reducing environmental and dietary exposure to OPs should be a priority for policymakers, parents, and health professionals working in rural, agricultural settings. Promoting organic diets could also be a practical strategy to reduce dietary pesticide exposure and associated health risks in these high-risk settings.

## Figures and Tables

**Table 1:** Socio-demographic and pesticide exposure among parents and preschool children.

Parameters		*n* (%) or Mean ± SD.
Parents		
Gender, *n* (%)	Male	55 (32.0)
	Female	117 (68.0)
Age, Mean ± SD.		50.1 ± 12.4
Monthly income, *n* (%)	<150 USD	114 (66.3)
	150–300 USD	36 (20.9)
	>300 USD	22 (12.8)
Education level, *n* (%)	Primary school or less	91 (52.9)
	Secondary school	58 (33.7)
	Bachelor’s degree or higher	23 (13.4)
Relationship to the primary caregiver, *n* (%)	Father/mother	66 (38.4)
	Legal guardians/family members ^a^	106 (61.6)
Occupation, *n* (%)	Non-farmers	31 (18.0)
	Farmers	141 (82.0)
Parental smoking, *n* (%)		33 (19.2)
Alcohol consumption, *n* (%)		76 (44.2)
Proximity of the household to farmland, *n* (%)	<0.5 km	22 (12.8)
	500 m–2 km	71 (41.3)
	>2–5 km	79 (45.9)
Children		
Children Age, Mean ± SD.		4.4 ± 0.5
Children gender, *n* (%)	Male	78 (45.3)
	Female	94 (54.7)
Children BMI, Mean ± SD.		15.6 ± 2.7
Playing on farmland, *n* (%)		86 (50.0)
Consumption of vegetables, *n* (%)	Never	15 (8.7)
	Rarely (1–2 servings/week)	93 (54.1)
	Often (3–5 servings/week)	56 (32.6)
	Always (6–7 servings/week)	8 (4.7)
Consumption of fruits, *n* (%)	Rarely (1–2 servings/week)	44 (25.6)
	Often (3–5 servings/week)	102 (59.3)
	Always (6–7 servings/week)	26 (15.1)
Usual place for purchasing vegetables and fruits, *n* (%)	Local markets	166 (96.5)
	Supermarkets	6 (3.5)

^a^ A person who was the primary caregiver, residing in the same household as the children and responsible for their care and upbringing.

**Table 2:** Urinary levels of DAP metabolites (ug/g creatinine) among preschool children.

Metabolites	%Detection	GM	Mean ± SD.	Median (P^25th^, P^75th^)
DMP	13.4	4.50	7.99 ± 13.30	7.47 (1.53, 7.47)
DMTP	10.5	1.41	8.43 ± 35.56	2.16 (0.44, 2.16)
DMDTP	5.8	0.59	0.82 ± 0.59	1.18 (0.24, 1.18)
DEP	38.4	2.45	12.16 ± 26.38	1.96 (0.40, 8.69)
DETP	34.9	5.20	10.46 ± 21.21	9.72 (1.99, 9.72)
DEDTP	1.2	0.89	1.18 ± 0.70	1.77 (0.36, 1.77)
Total DMP	23.3	7.43	17.24 ± 38.06	10.81 (2.21, 10.81)
Total DEP	60.5	10.51	23.81 ± 42.84	13.46 (2.87, 19.28)
Total DAP	67.4	20.58	41.07 ± 57.66	24.26 (6.17, 46.21)

Total DMP = DMP + DMTP + DEDTP; Total DEP = DEP + DETP + DEDTP; Total DAP = total DMP + total DEP.

**Table 3:** Neurobehavioral performance among preschool children.

Neurobehavioral Tests		Mean ± SD.	Median (P^25th^, P^75th^)	Min–Max
PEG	Dominant hand	7.48 ± 1.62	8 (6, 9)	3–11
	Preferred hand	6.85 ± 1.56	7 (6, 8)	1–10
	Both hands	5.52 ± 1.78	5 (4, 7)	0–9
DST	Forward digit span	7.03 ± 1.98	7 (6, 9)	2–11
	Maximum digit span	4.79 ± 1.17	5 (4, 6)	2–6
OMT	Immediate recall	14.64 ± 1.32	15 (14, 15)	11–16
	Delay recall	14.85 ± 1.18	15 (14, 15)	12–16
	Recognition	14.03 ± 1.60	15 (12, 15)	12–15
VMI	Raw score	11.48 ± 3.19	12 (10, 13.75)	1–19
	Standard score	99.79 ± 14.06	103 (92, 107)	65–142

PEG = Purdue pegboard test; DST = digit span test; OMT = object memory test; VMI = visual-motor integration.

**Table 4:** Multiple linear regression for investigating factors associated with urinary DAP metabolites among preschool children.

Factors	Ln Total DMP			Ln Total DEP		
	Beta	95% CI	*p* Value	Beta	95% CI	*p* Value
Parental factors						
Parental age	−0.004	−0.0.31, 0.024	0.792	0.003	−0.026, 0.032	0.820
Parental gender	−0.041	−0.416, 0.027	0.860	0.089	−0.393, 0.570	0.717
Parental smoking	0.052	−0.510, 0.614	0.855	−0.115	−0.706, 0.477	0.702
Parental farmer status	0.730	0.138, 1.322	0.016 *	0.668	0.044, 1.291	0.036 *
Parental relationship	0.370	−0.312, 1.052	0.286	−0.119	−0.837, 0.599	0.745
Proximity of household to farmland	−0.039	−0.307, 0.230	0.777	−0.083	−0.366, 0.199	0.561
Children factors						
Children gender	−0.045	−0.407, 0.317	0.807	0.117	−0.264, 0.498	0.546
Children BMI	−0.071	−0.139, −0.004	0.039 *	−0.049	−0.120,0.022	0.172
Playing on farmland	0.267	−0.119, 0.653	0.174	0.265	−0.141, 0.672	0.200
Frequency of vegetable consumption	0.001	−0.273, 0.276	0.994	−0.264	−0.553, 0.025	0.073
Frequency of fruit consumption	0.242	−0.077, 0.560	0.136	0.398	0.063, 0.733 *	0.020 *

* *p* value < 0.05.

**Table 5:** Multiple linear regression for investigating urinary levels of DAP metabolites associated with neurobehavioral performance in preschool children.

Outcomes		Ln Total DMP Levels		Ln Total DEP Levels	
		Beta (95% CI)	*p* Value	Beta (95% CI)	*p* Value
PEG	Dominant hand	0.211 (−0.034, 0.457)	0.091	−0.428 (−0.661, −0.194)	<0.001 **
	Preferred hand	0.143 (−0.096, 0.381)	0.239	−0.376 (−0.603, −0.149)	0.001 **
	Both hands	0.104 (−0.157, 0.366)	0.431	−0.524 (−0.773, −0.276)	<0.001 **
DST	Forward digit span	−0.105 (−0.393, 0.184)	0.474	−0.102 (−0.377, 0.173)	0.464
	Maximum digit span	−0.033 (−0.204, 0.138)	0.707	−0.081 (0.243, 0.082)	0.330
OMT	Immediate recall	−0.086 (−0.282, 0.110)	0.387	−0.053 (−0.240, 0.133)	0.573
	Delay recall	−0.004 (−0.187, 0.179)	0.967	−0.025 (−0.199, 0.149)	0.773
	Recognition	0.013 (−0.237, 0.263)	0.917	−0.067 (−0.305, 0.171)	0.573
VMI	Raw score	−0.285 (−0.772, 0.203)	0.250	−0.090 (−0.554, 0.374)	0.703
	Standard score	−1.113 (−3.159, 0.933)	0.284	−0.730 (−2.677, 1.218)	0.461

Adjusted with confounders, including parental age, parental factors (gender, education, monthly income, smoking, and alcohol consumption) and child factors (age, gender, and BMI); PEG = Purdue pegboard; DST = digit span test; OMT = object memory test; VMI = visual motor integration; ** *p* value < 0.01.

## Data Availability

The original contributions presented in the study are included in the article. Further inquiries can be directed to the corresponding author.

## Supplementary Materials

The following supporting information can be downloaded at: https://www.mdpi.com/article/10.3390/toxics12120855/s1, Table S1. The association between parental occupation and children’s frequency of vegetable and fruit consumption; Table S2. The association between children’s BMI and frequency of fruit and vegetable consumption

## References

[1] Buralli R.J., Marques R.C., Dórea J.G. (2023). Pesticide effects on children’s growth and neurodevelopment. Curr. Opin. Environ. Sci. Health.

[2] Etzel R.A., Landrigan P.J. (2024). Children’s exquisite vulnerability to environmental exposures. Textbook of Children’s Environmental Health.

[3] International Trade Administration (2024). Thailand—Country Commercial Guide. https://www.trade.gov/country-commercial-guides/thailand-agriculture.

[4] Vanwambeke S.O., Somboon P., Lambin E.F. (2007). Rural transformation and land use change in northern Thailand. J. Land Use Sci..

[5] Laohaudomchok W., Nankongnab N., Siriruttanapruk S., Klaimala P., Lianchamroon W., Ousap P., Jatiket M., Kajitvichyanukul P., Kitana N., Siriwong W. (2021). Pesticide use in Thailand: Current situation, health risks, and gaps in research and policy. Hum. Ecol. Risk Assess..

[6] Suarez-Lopez J.R., Nazeeh N., Kayser G., Suárez-Torres J., Checkoway H., López-Paredes D., Jacobs D.R., Cruz F. (2020). Residential proximity to greenhouse crops and pesticide exposure (via acetylcholinesterase activity) assessed from childhood through adolescence. Environ. Res..

[7] Roberts J.R., Karr C.J. (2012). Council on Environmental Health. Pesticide exposure in children. Pediatrics.

[8] Šulc L., Figueiredo D., Huss A., Kalina J., Gregor P., Janoš T., Šenk P., Dalecká A., Andrýsková L., Kodeš V. (2023). Current-use pesticide exposure pathways in Czech adults and children from the CELSPAC-SPECIMEn cohort. Environ. Int..

[9] Ahmad M.F., Ahmad F.A., Alsayegh A.A., Zeyaullah M., AlShahrani A.M., Muzammil K., Saati A.A., Wahab S., Elbendary E.Y., Kambal N. (2024). Pesticides impacts on human health and the environment with their mechanisms of action and possible countermeasures. Heliyon.

[10] Sapbamrer R., Hongsibsong S., Khacha-Ananda S. (2020). Urinary organophosphate metabolites and oxidative stress in children living in agricultural and urban communities. Environ. Sci. Pollut. Res. Int..

[11] Alkon A., Gunier R.B., Hazard K., Castorina R., Hoffman P.D., Scott R.P., Anderson K.A., Bradman A. (2022). Preschool-age children’s pesticide exposures in child care centers and at home in Northern California. J. Pediatr. Health Care.

[12] Dórea J.G. (2021). Exposure to environmental neurotoxic substances and neurodevelopment in children from Latin America and the Caribbean. Environ. Res..

[13] Sapbamrer R., Hongsibsong S. (2019). Effects of prenatal and postnatal exposure to organophosphate pesticides on child neurodevelopment in different age groups: A systematic review. Environ. Sci. Pollut. Res. Int..

[14] González-Alzaga B., Romero-Molina D., López-Flores I., Giménez-Asensio M.J., Hernández A.F., Lacasaña M. (2020). Urinary levels of organophosphate pesticides and predictors of exposure in pre-school and school children living in agricultural and urban communities from south Spain. Environ. Res..

[15] Wager J.L., Thompson J.A. (2024). Development and child health in a world of synthetic chemicals. Pediatr. Res..

[16] Prapamontol T., Sutan K., Laoyang S., Hongsibsong S., Lee G., Yano Y., Hunter R.E., Ryan P.B., Barr D.B., Panuwet P. (2014). Cross-validation of gas chromatography-flame photometric detection and gas chromatography-mass spectrometry methods for measuring dialkylphosphate metabolites of organophosphate pesticides in human urine. Int. J. Hyg. Environ. Health.

[17] Hornung R.W., Reed L.D. (1990). Estimation of average concentration in the presence of nondetectable values. Appl. Occup. Environ. Hyg..

[18] Rohlman D.S., Lasarev M., Anger W.K., Scherer J., Stupfel J., McCauley L. (2007). Neurobehavioral performance of adult and adolescent agricultural workers. Neurotoxicology.

[19] Rohlman D.S., Lucchini R., Anger W.K., Bellinger D.C., van Thriel C. (2008). Neurobehavioral testing in human risk assessment. Neurotoxicology.

[20] Rohitrattana J., Siriwong W., Suittiwan P., Robson M.G., Strickland P.O., Rohlman D.S., Fiedler N. (2014). Adaptation of a neurobehavioral test battery for Thai children. Rocz. Państwowego Zakładu Hig..

[21] Beery K.E., Beery N.A. (2004). The Beery-Buktenica Developmental Test of Visual-Motor Integration (Manual).

[22] Spencer T.D., Kruse L., Volkmar F.R. (2013). Beery-Buktenica developmental test of visual-motor integration. Encyclopedia of Autism Spectrum Disorders.

[23] Thongjan N., Prapamontol T., Liwsrisakun C., Chairuangsri S., Hongsibsong S., Norbäck D. (2024). Organophosphate insecticide exposure and respiratory symptoms among school children in Northern Thailand: Interaction by biomass burning, dampness, and season. Sci. Total Environ..

[24] Wongta A., Sawang N., Tongjai P., Jatiket M., Hongsibsong S. (2022). The assessment of organophosphate pesticide exposure among school children in four regions of Thailand: Analysis of dialkyl phosphate metabolites in students’ urine and organophosphate pesticide residues in vegetables for school lunch. Toxics.

[25] The Office of Agricultural Regulation Department, Department of Agriculture (2024). Statistics of Hazardous Chemicals Imported into Thailand. https://www.doa.go.th/ard/?page_id=386.

[26] Holme F., Thompson B., Holte S., Vigoren E.M., Espinoza N., Ulrich A., Griffith W., Faustman E.M. (2016). The role of diet in children’s exposure to organophosphate pesticides. Environ. Res..

[27] Pududu B.A., Rother H.A. (2021). Whose jurisdiction is home contamination? Para-occupational ‘take-home’ herbicide residue exposure risks among forestry workers’ families in South Africa. Int. J. Environ. Res. Public Health.

[28] Kalweit A., Herrick R.F., Flynn M.A., Spengler J., Berko J.K., Levy J.I., Ceballos D.M. (2020). Eliminating take-home exposures: Recognizing the role of occupational health and safety in broader community health. Ann. Work. Expo. Health.

[29] Khan K.M., Gaine M.E., Daniel A.R., Chilamkuri P., Rohlman D.S. (2024). Organophosphorus pesticide exposure from house dust and parent-reported child behavior in Latino children from an orchard community. Neurotoxicology.

[30] Simaremare S.R.S., Hung C.C., Yu T.H., Hsieh C.J., Yiin L.M. (2021). Association between pesticides in house dust and residential proximity to farmland in a rural region of Taiwan. Toxics.

[31] Butler-Dawson J., Galvin K., Thorne P.S., Rohlman D.S. (2016). Organophosphorus pesticide exposure and neurobehavioral performance in Latino children living in an orchard community. Neurotoxicology.

[32] López-Gálvez N., Wagoner R., Quirós-Alcalá L., Ornelas Van Horne Y., Furlong M., Avila E., Beamer P. (2019). Systematic literature review of the take-home route of pesticide exposure via biomonitoring and environmental monitoring. Int. J. Environ. Res. Public Health.

[33] Carroquino M.J., Posada M., Landrigan P.J. (2012). Environmental toxicology: Children at risk. Environ. Toxicol..

[34] Kordas K., Cantoral A., Desai G., Halabicky O., Signes-Pastor A.J., Tellez-Rojo M.M., Peterson K.E., Karagas M.R. (2022). Dietary exposure to toxic elements and the health of young children: Methodological considerations and data needs. J. Nutr..

[35] Xu W., Dong Y., Liu S., Hu F., Cai Y. (2024). Association between organophosphorus pesticides and obesity among American adults. Environ. Health.

[36] Siripunyo T. (2017). Determination of organophosphate pesticides residues in fruits, vegetables and health risk assessment among consumers in Chiang Mai Province, Northern Thailand. Res. J. Environ. Toxicol..

[37] Kähkönen K., Sandell M., Rönkä A., Hujo M., Nuutinen O. (2021). Children’s fruit and vegetable preferences are associated with their mothers’ and fathers’ preferences. Foods.

[38] Guzman-Torres H., Sandoval-Pinto E., Cremades R., Ramírez-de-Arellano A., García-Gutiérrez M., Lozano-Kasten F., Sierra-Díaz E. (2023). Frequency of urinary pesticides in children: A scoping review. Front. Public Health.

[39] Bradman A., Quirós-Alcalá L., Castorina R., Schall R.A., Camacho J., Holland N.T., Barr D.B., Eskenazi B. (2015). Effect of organic diet intervention on pesticide exposures in young children living in low-income urban and agricultural communities. Environ. Health Perspect..

[40] van Wendel de Joode B., Mora A.M., Lindh C.H., Hernández-Bonilla D., Córdoba L., Wesseling C., Hoppin J.A., Mergler D. (2016). Pesticide exposure and neurodevelopment in children aged 6–9 years from Talamanca, Costa Rica. Cortex.

[41] Kofman O., Berger A., Massarwa A., Friedman A., Jaffar A.A. (2006). Motor inhibition and learning impairments in school-aged children following exposure to organophosphate pesticides in infancy. Pediatr. Res..

[42] Tsai Y.H., Lein P.J. (2021). Mechanisms of organophosphate neurotoxicity. Curr. Opin. Toxicol..

[43] Jayasinghe S.S., Pathirana K.D., Buckley N.A. (2012). Effects of acute organophosphorus poisoning on function of peripheral nerves: A cohort study. PLoS ONE.

[44] Karami-Mohajeri S., Nikfar S., Abdollahi M. (2014). A systematic review on the nerve–muscle electrophysiology in human organophosphorus pesticide exposure. Hum. Exp. Toxicol..

[45] Muñoz-Quezada M.T., Lucero B.A., Barr D.B., Steenland K., Levy K., Ryan P.B., Iglesias V., Alvarado S., Concha C., Rojas E. (2013). Neurodevelopmental effects in children associated with exposure to organophosphate pesticides: A systematic review. Neurotoxicology.

[46] Vester A., Caudle W.M. (2016). The synapse as a central target for neurodevelopmental susceptibility to pesticides. Toxics.

[47] Farkhondeh T., Mehrpour O., Forouzanfar F., Roshanravan B., Samarghandian S. (2020). Oxidative stress and mitochondrial dysfunction in organophosphate pesticide-induced neurotoxicity and its amelioration: A review. Environ. Sci. Pollut. Res. Int..

[48] Naughton S.X., Terry A.V. (2018). Neurotoxicity in acute and repeated organophosphate exposure. Toxicology.

