# Supplementary material for: Neurobehavioral Performance in Preschool Children Exposed Postnatally to Organophosphates in Agricultural Regions, Northern Thailand
Source: Toxics. 2024 Nov 27;12(12):855. doi: 10.3390/toxics12120855 (PMC11679742; doi:10.3390/toxics12120855)
Supplement: Supplementary file 1 [file toxics-12-00855-s001.zip › toxics-3311499-supplementary.pdf]

Table S1. The association between parental occupation and children's frequency of vegetable and fruit consumption.

**Paramters**		**Parental occupation**		***p* value**
		**Farmers**	**Non-farmers**	
children's frequency of vegetable consumption	Never	13 (9.2%)	2 (6.5%)	0.030*
	Rarely (1–2 servings/week)	69 (48.9%)	24 (77.4%)	
	Often (3–5 servings/week)	51 (36.2%)	5 (16.1%)	
	Always (6–7 servings/week)	8 (5.7%)	0 (0%)	
children's frequency of fruit consumption	Rarely (1–2 servings/week)	25 (17.7%)	19 (61.3%)	<0.001**
	Often (3–5 servings/week)	90 (63.8%)	12 (38.7%)	
	Always (6–7 servings/week)	26 (18.4%)	0 (0%)	

* *p* value <0.05; ** *p* value <0.01

Table S2. The association between children's BMI and frequency of fruit and vegetable consumption.

Parameters		n	Mean±SD. of BMI	*p* value
children's frequency of vegetable consumption	Never	15	15.02±1.89	0.532
	Rarely (1–2 servings/week)	93	15.64±1.96	
	Often (3–5 servings/week)	54	15.59±3.55	
	Always (6–7 servings/week)	8	16.74±3.79	
children's frequency of fruit consumption	Rarely (1–2 servings/week)	44	15.39±2.21	0.623
	Often (3–5 servings/week)	100	15.79±2.86	
	Always (6–7 servings/week)	26	25.37±2.52	
